# Affimer proteins for F-actin: novel affinity reagents that label F-actin in live and fixed cells

**DOI:** 10.1038/s41598-018-24953-4

**Published:** 2018-04-26

**Authors:** Anna Lopata, Ruth Hughes, Christian Tiede, Sarah M. Heissler, James R. Sellers, Peter J. Knight, Darren Tomlinson, Michelle Peckham

**Affiliations:** 1 School of Molecular and Cellular Biology, Faculty of Biological Sciences, University of Leeds, Leeds, UK; 2 Astbury Centre for Structural Molecular Biology, University of Leeds, Leeds, UK; 3 Laboratory of Molecular Physiology, National Heart, Lung, and Blood Institute, Bethesda, MD 20892 USA

## Abstract

Imaging the actin cytoskeleton in cells uses a wide range of approaches. Typically, a fluorescent derivative of the small cyclic peptide phalloidin is used to image F-actin in fixed cells. Lifeact and F-tractin are popular for imaging the cytoskeleton in live cells. Here we characterised novel affinity reagents called Affimers that specifically bind to F-actin *in vitro* to determine if they are suitable alternatives as eGFP-fusion proteins, to label actin in live cells, or for labeling F-actin in fixed cells. *In vitro* experiments showed that 3 out of the 4 Affimers (Affimers 6, 14 and 24) tested bind tightly to purified F-actin, and appear to have overlapping binding sites. As eGFP-fusion proteins, the same 3 Affimers label F-actin in live cells. FRAP experiments suggest that eGFP-Affimer 6 behaves most similarly to F-tractin and Lifeact. However, it does not colocalise with mCherry-actin in dynamic ruffles, and may preferentially bind stable actin filaments. All 4 Affimers label F-actin in methanol fixed cells, while only Affimer 14 labels F-actin after paraformaldehyde fixation. eGFP-Affimer 6 has potential for use in selectively imaging the stable actin cytoskeleton in live cells, while all 4 Affimers are strong alternatives to phalloidin for labelling F-actin in fixed cells.

## Introduction

The actin cytoskeleton is widely visualised in cell biology, owing to the role it plays in cellular processes, including intracellular transport, migration, cell shape and gene regulation. Although many reagents exist for imaging actin, it still is a challenge to visualise certain actin structures^1^. In fixed cells, typically fluorescent derivatives of phalloidin are used^2^. Phalloidin is a small bicyclic peptide (M_r_ 1,250) isolated from Amanita phalloides^3^. Fluorescent derivatives of phalloidin bind to F-actin with high affinity (Kd ~0.27 μM)^2^. X-ray fiber diffraction and mutagenesis analysis suggest that phalloidin is located in the contact region of three actin subunits, and helps to stabilise F-actin by binding between the two long-pitch strands of the actin filament^4,5^. The stability of F-actin induced by binding of phalloidin propagates ~7 actin subunits along the filament from the binding site^6^. The drawback of phalloidin is that it is less useful in live cells. The dissociation rate of fluorescent phalloidin to cellular F-actin filaments is very slow (half time ~400 s^7^), which may account for the observation that it can result in the formation of stable actin aggregates^8^ unless used at very low concentrations^7,9^. Moreover, it does not bind to all F-actin structures *in vivo*^10^.

In live cells, there is a wide choice of probes to visualise actin. These include directly labelling actin (actin labelled with rhodamine, or eGFP-actin^11,12^), the addition of cell penetrating actin-binding peptides and the expression of actin-binding protein reagents. Visualizing actin by expressing eGFP-actin has been used for some time^12^ but has numerous drawbacks. As both G-actin and F-actin are labelled, there is a higher fluorescent background from non-filamentous actin^13^, and expression of eGFP-actin^14^ can affect cell behaviour^15^ and see a recent review^1^. This has led to the development of several fluorescent F-actin-binding peptides (e.g. LifeAct and F-tractin) to avoid this problem. Lifeact consists of eGFP fused to the C-terminus of the first 17 amino acids from the yeast actin-binding protein Abp140^16^. It binds to F-actin with a Kd of 2.2 ± 0.3 μM. However, its binding affinity for G-actin is 10-fold higher than for F-actin, which can result in background staining in live cells^16^. Despite this, it has been reported to enable a clearer visualisation of the actin cytoskeleton than eGFP-actin^17^, although it does not recognize all actin structures in cells (reviewed in^1^). F-tractin consists of eGFP fused to the N-terminus of an F-actin-binding peptide, 44 amino acids long (residues 9–52), from the rat inositol 1,4,5-trisphosphate 3-kinase (ITPK^18^). It binds to F-actin with a Kd of ~10 μM^19^, and works well in labelling F-actin in cells^20^ showing an actin organization similar to that reported by phalloidin^21^. The actin-binding CH domains of utrophin, in which eGFP is fused to the N-terminus, have also been used. The CH domains bind to actin with a Kd of ~18 μM^22^. However, this probe can have strong effects on actin dynamics^20^.

Two less well characterised reagents have been recently developed to label F-actin in live cells. The first uses a nanobody to actin fused to eGFP (reviewed in^1^), but its behaviour in cells has not been extensively studied. The second, SiR-actin, is derived from the F-actin binding product jasplakinolide^23^. While SiR-actin has the advantage that it is cell-permeable, it has the disadvantage that it only emits in the far-red, and it is unclear if it may also affect actin dynamics.

Here we have explored novel, synthetic, actin binding proteins isolated from a phage library screen of ‘Affimers’^24,25^ to attempt to find a new protein that would be useful in labelling the actin cytoskeleton in both live and fixed cells. Affimers are non-antibody-based affinity reagents, based on the plant-derived phytocystatins, which are small (~100 aa) protein inhibitors of cysteine proteases^25^. The two loops that contain inhibitory sequences in the consensus phytocystatin sequence, have been replaced with two loops of nine amino acid (excluding cysteine). A wide range of Affimers have now been identified, through a phage library screen to a range of targets, and have been shown to have a wide range of uses^24,26–29^. Screening against F-actin, we recovered 4 Affimers, and here we characterize their ability to bind actin, both *in vitro* and *in vivo* in live and fixed cells to provide an alternative versatile reagent to visualize the actin cytoskeleton. We additionally demonstrate the potential ability to further isolate Affimers capable of recognizing different forms of actin.

## Results and Discussion

### Three unique Affimers, 6, 14 and 24, bind strongly to F-actin *in vitro*

**Figure 1:**
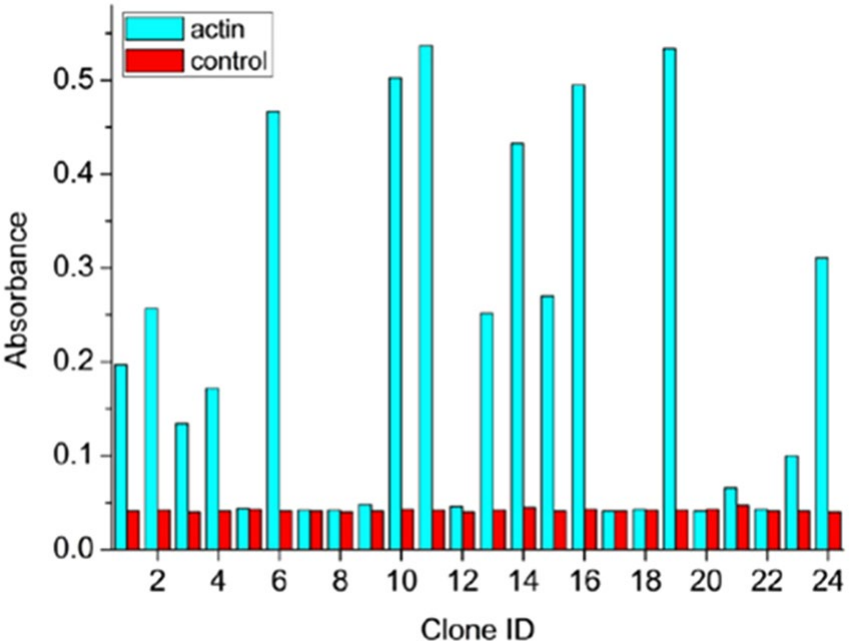
Screening of the Affimer clones for actin binding using phage ELISA. 24 clones were tested in wells coated with actin, and in wells containing only buffer. Absorbance of oxidised TMB at 620 nm was recorded. Clones were sequenced and those with unique sequences, and with the highest level of signal above background (clones 2, 6, 14 and 24) were selected for further analysis. All other clones that showed affinity for actin were identical to one or other of the selected clones.

Screening of the Affimer phage-display library allowed us to recover many Affimers that bound F-actin as shown by ELISA (Fig. 1). Sequencing of the clones obtained showed that of these binders, there were 4 unique Affimer sequences (Affimers 2, 6, 14 and 24). We went on to test these in more detail by subcloning them into a bacterial expression vector for expression and purification.

**Figure 2:**
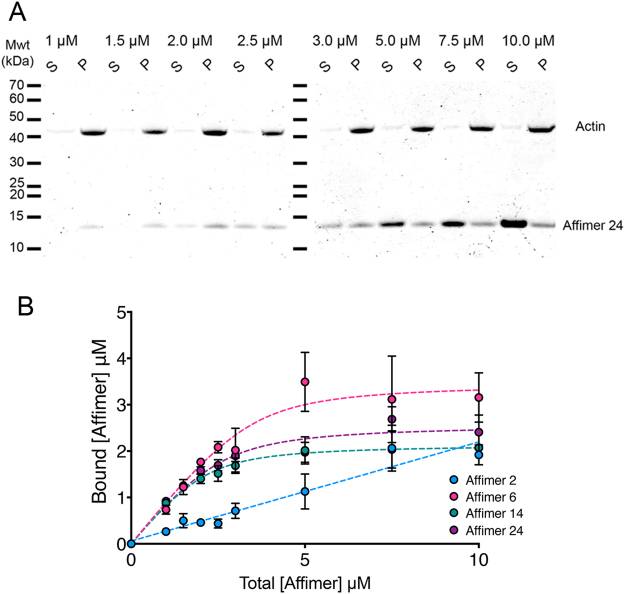
Binding of Affimers to F-actin *in vitro*. (**A**) Shows example gels from one of the experiments with Affimer 24 in which increasing concentrations of Affimer were mixed with 2.5μM F-actin. Lanes show supernatant (S) and pellet (P) from sedimentation of the mixtures. (**B**) Shows the mean values from three separate experiments, together with the standard error. Dashed lines indicate the quadratic fits to the data for Affimers 6, 14 and 24. The dashed line for Affimer 2 is a linear fit.

The binding of the purified Affimers to F-actin *in vitro* was tested using a sedimentation assay taking advantage of the ability for actin filaments to be pelleted in the ultracentrifuge at 110,000 *g* (Fig. 2A). Affimers 6, 14 and 24 bound strongly to F-actin, with 1:1 stoichiometry, and with Kds of 0.31 ± 0.17, 0.30 ± 0.05 and 0.38 ± 0.14 μM for Affimer 6, Affimer 14 and Affimer 24, respectively. These values are similar to that measured for fluorescently labelled phalloidin (0.27 μM)^2^. In contrast, Affimer 2 bound to F-actin very weakly (Fig. 2B).

### Strongly binding Affimers appear to bind similarly to F-actin

**Figure 3:**
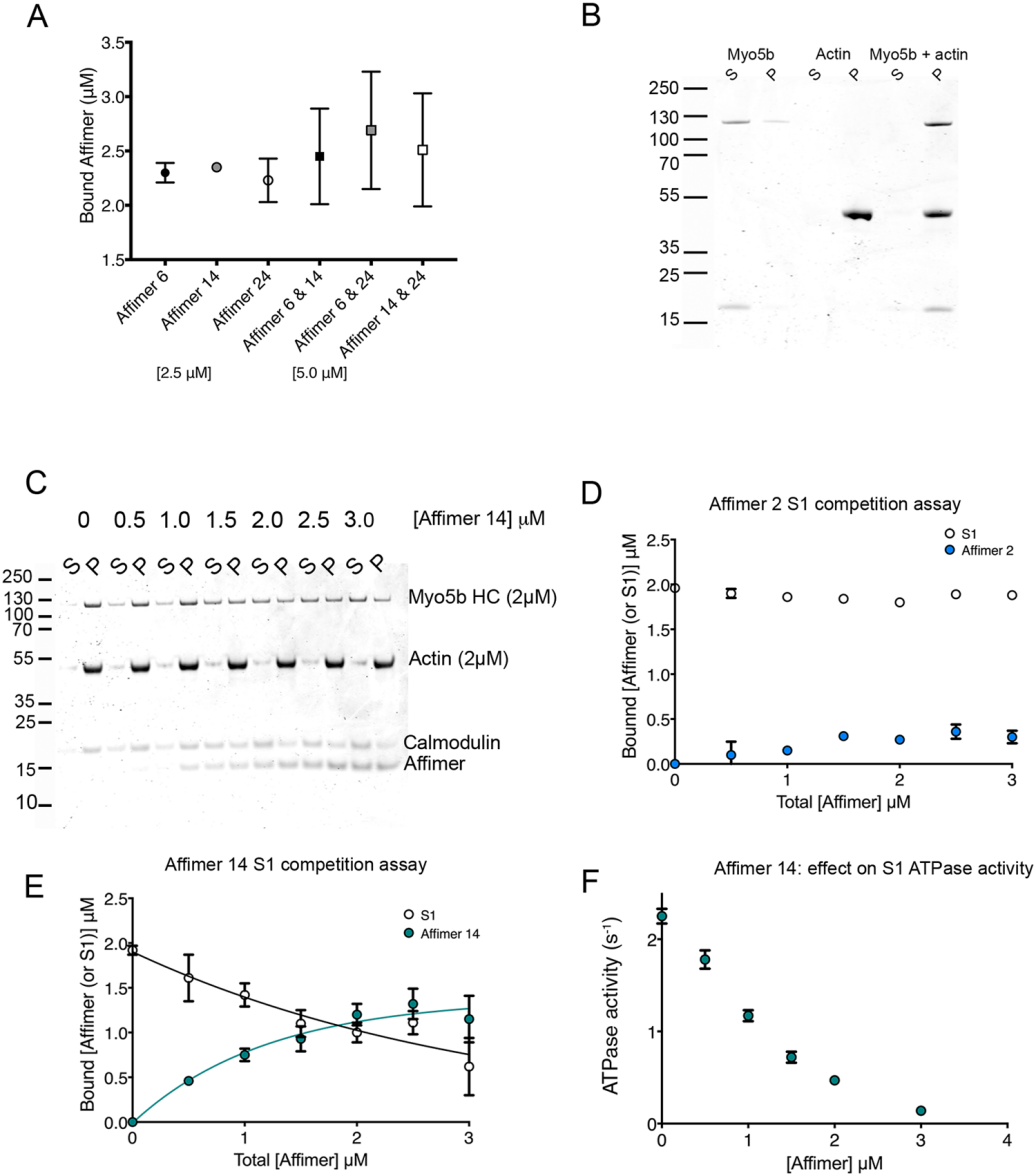
Competition of Affimers with myosin and each other for binding to F-actin. (**A**) Competition assay for combinations of 2 Affimers. The values shown are the mean of three separate experiments together with the standard deviation. (**B**) 2.5 µM Myo5b S1 (subfragment 1) co-sediments with 2.5 µM actin in the absence of ATP. Lanes show Myo5b S1 (supernatant (S) and pellet (P)), actin (S and P) and the combination of Myo5b S1 and actin. (**C**) An example SDS PAGE gel from a competition experiment between 2 μM Myo5b S1 and 0–3 μM Affimer 14 for binding to 2 μM F-actin. HC indicates the position of the heavy chain of Myo5b S1. (**D**) Results for the competition assay between Myo5b S1 and Affimer 2. (**E**) Competition assay results for Myo5b S1 and Affimer 14. (**F**) Effects of increasing Affimer 14 concentration on Myo5b S1 ATPase activated by 4 μM F-actin.

To try to determine where the Affimers bind on actin, two competition assays were performed. First, mixtures of Affimers were added to F-actin to determine if the Affimers competed for a common binding site on actin. Affimer 2 was not tested in these experiments as it only bound weakly. We found that only ~2.5 μM Affimer is bound to 2.5 μM F-actin, in all of the conditions, including mixtures with higher starting concentrations of Affimers (Fig. 3A). These experiments demonstrate that the binding sites for each Affimer to F-actin must be mutually exclusive, as increasing the number of Affimers in the mixtures did not increase the amount of Affimer that co-sedimented with F-actin.

Second, we tested the ability of Myo5b S1 to bind to actin in the presence of the Affimer (Fig. 3B–F). These experiments were performed with the weak binding Affimer (Affimer 2) and one of the strong binding Affimers (Affimer 14), as the experiment above demonstrated that Affimers 6, 14 and 24 bind to overlapping binding sites on F-actin. Myo5b is found in the pellet with actin, when no Affimers are present (Fig. 3B). If Affimers bind to the same site as myosin, increasing amounts of Affimer will reduce the amount of myosin that pellets (Fig. 3C). Affimer 2 did not affect the ability of Myo5b to bind to F-actin (Fig. 3D), consistent with its weak binding to actin. However, Affimer 14 decreased the binding of Myo5b to actin (Fig. 3E). Under the conditions used here (~200 mM salt), binding of Myo5b is likely to be relatively weak, and the ability of Affimer 14 to displace myosin suggests it binds via a hydrophobic interaction with the actin filament. We explored this further by testing the effect of Affimer 14 on the actin-activated ATPase activity of Myo5b. Increasing concentrations of Affimer 14 almost completely abolished the actin-activated ATPase (Fig. 3F). This suggests that Affimer 14 can interfere with the binding of Myo5b S1ADPP_i_ to F-actin under the conditions used here. High resolution structures of F-actin complexed with either human non-muscle myosin-2C^30^, *Dictyostelium* myosin-1E^31^, or chicken myosin-5a^32^ show that the same parts of actin interact with very different myosins. Thus, the results we have obtained here using Myo5b are likely to apply to other myosins too.

### Three eGFP-Affimers label F-actin in live cells

**Figure 4:**
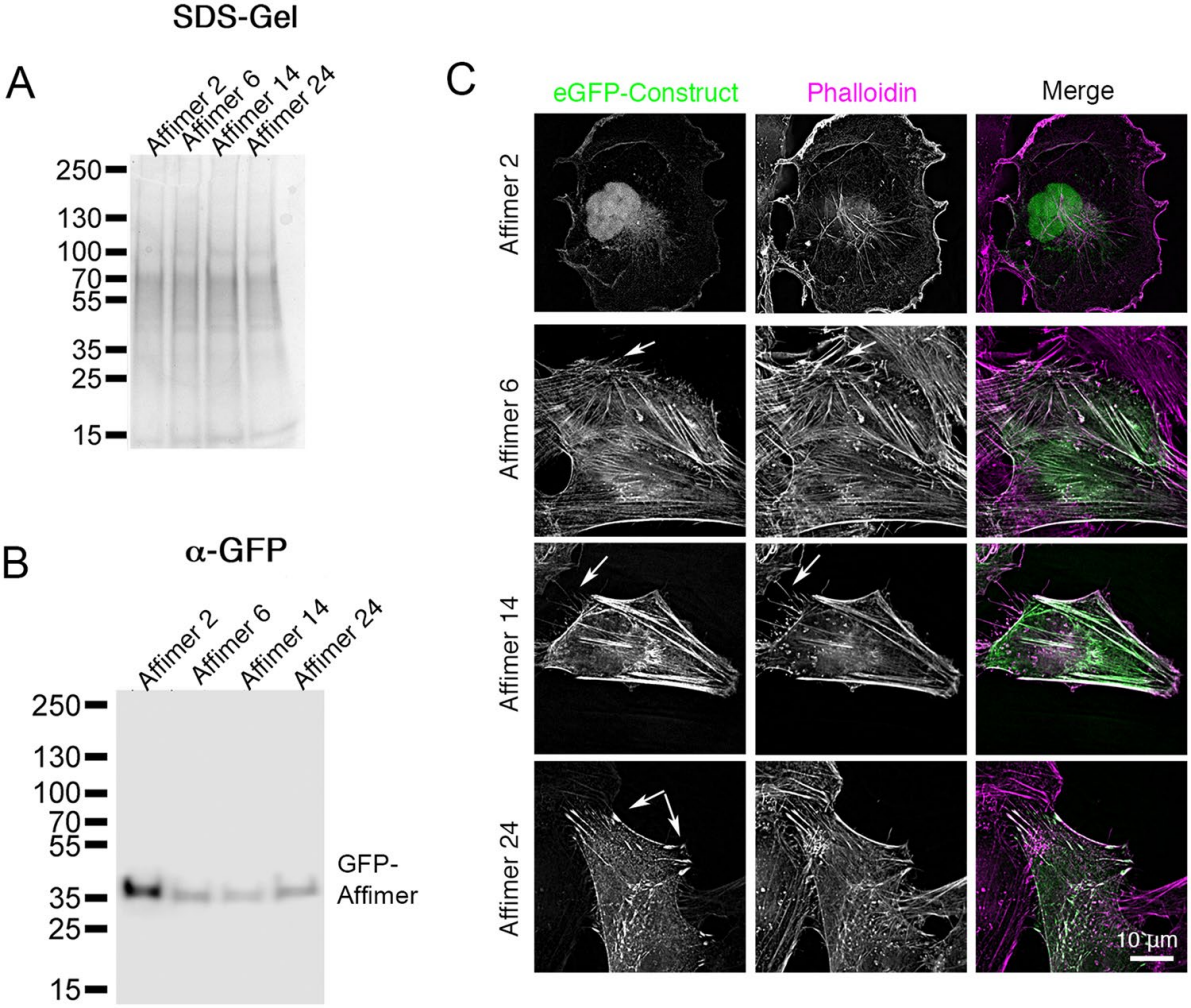
eGFP-Affimers 6, 14 and 24 label F-actin. (**A**) SDS PAGE gels of protein samples (to show approximately equivalent loading of samples) from transfected cells as indicated, and associated western blot (**B**) using an antibody for eGFP. (**C**) Fluorescent images of fixed Hela cells expressing fluorescent eGFP-Affimer constructs (green in merged image), co-stained with fluorescent phalloidin (magenta in merged image). Images were captured using the Deltavision deconvolution microscope. Arrows for Affimer 6 and 14 show weak staining of thin protrusions by the eGFP-Affimer compared to phalloidin. Arrows for Affimer 24 show the accumulation of Affimer 24 in focal adhesions. Scale bar as shown.

We next tested if Affimers could label F-actin in live cells, by fusing the sequence for eGFP to the N-terminus of the Affimer sequences, and transfecting the cells with the eGFP-fusion proteins. Western blotting showed that all the Affimers were expressed at approximately similar overall levels, and were the expected sizes (Fig. 4A,B). eGFP-Affimers 6, 14 and 24 all labelled the F-actin cytoskeleton (Fig. 4C) but eGFP-Affimer 2 did not, consistent with its weak binding affinity to F-actin *in vitro*.

Interestingly, the pattern of localisation did not appear to be identical for the three eGFP-Affimers. Thus, although we found that the binding sites for F-actin for each of the Affimers was mutually exclusive, it is unlikely that they bind to actin in an identical manner. Affimer 6 appeared to localise well to actin structures throughout the cell, with finely detailed structures evident (Fig. 4B). Affimer 14 and 24 either were more likely to associate with bundled actin, or were more likely to induce actin bundling, with Affimer 24 demonstrating higher levels of staining in structures that appeared to be focal adhesions. However, the localisation of GFP-Affimers and phalloidin in these cells was very similar, with the exception that fine protrusions were not well labelled by the eGFP-Affimers. This initial set of experiments on Hela cells suggested to us that the eGFP-Affimers may preferentially bind to a subset of actin filaments in the cell, and that Affimers 14 and 24 may be altering actin organization somewhat, as reported for other F-actin reporters^1,20,21^.

**Figure 5:**
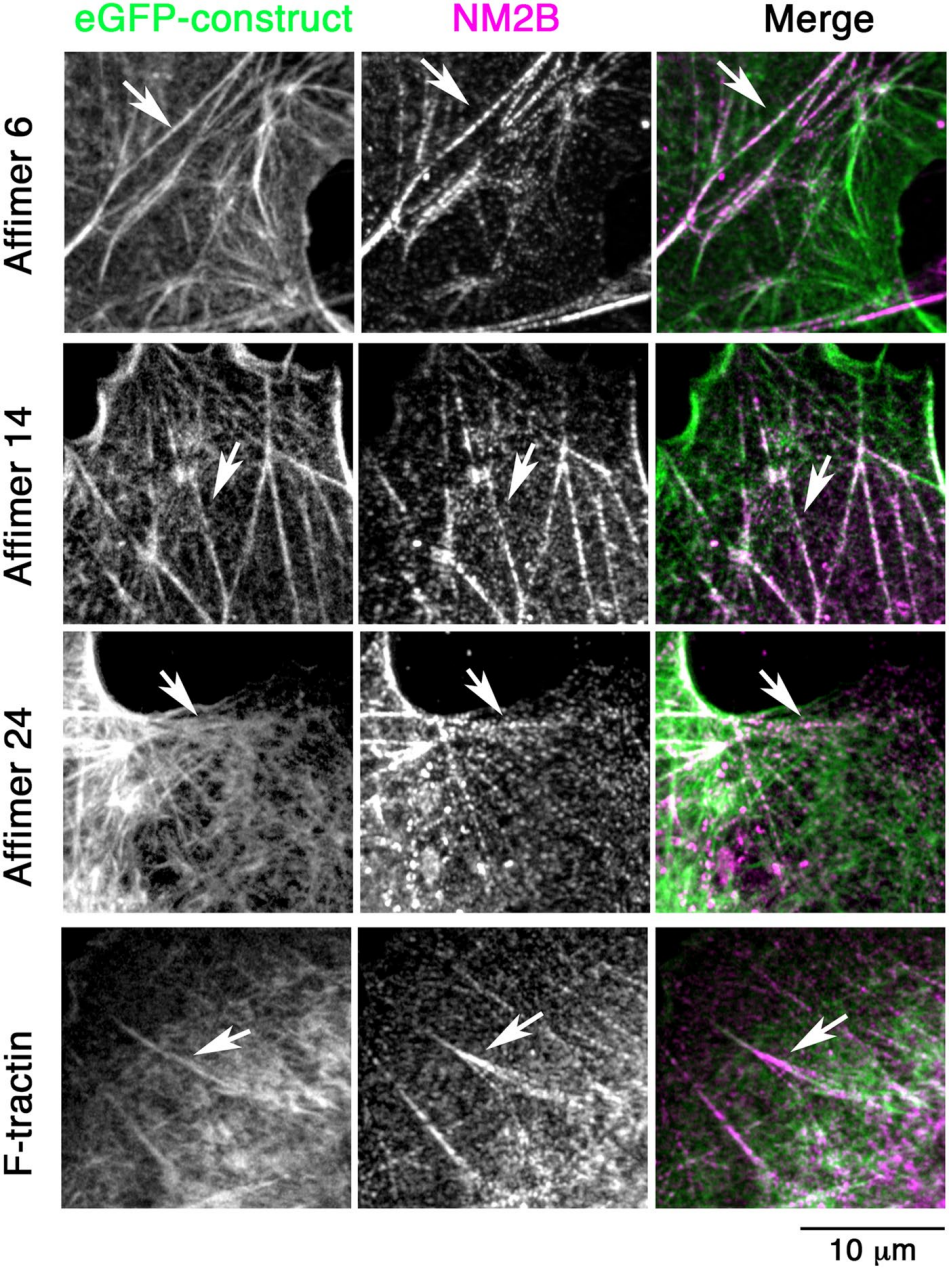
Co-staining of non-muscle myosin 2B (NM2B, in magenta in merged image) with eGFP-Affimers or F-tractin (green in merged image) in Hela cells. Images captured using the Zeiss 880 LSM confocal microscope. Arrows show co-localisation of NM2B with actin filaments. Scale bar as shown.

As the *in vitro* experiments suggested that when F-actin is saturated with Affimers, binding of myosin S1 to F-actin could be inhibited, we tested if expression of eGFP-Affimers affected the co-localisation of non-muscle myosin with F-actin in cells. Fixing and co-staining for NM2B (the main isoform of non-muscle myosin expressed in COS-7 cells) showed that NM2B was associated with F-actin structures decorated by eGFP-Affimers (Fig. 5). This suggests that, at the levels of expression of the Affimers tested, NM2B was still recruited to F-actin structures in the cells, although we have not quantified this.

**Figure 6:**
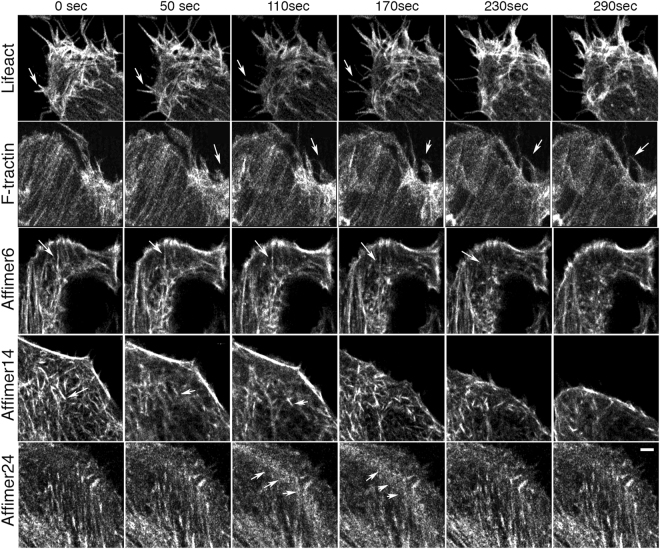
Stills from time-lapse movies for the eGFP-Affimers, F-tractin and Lifeact in live B16 cells. Time shown is in seconds. Arrows show small thin protrusions (for F-tractin and Lifeact). Arrows in panels for Affimer 6 indicate cellular regions with dynamic actin filament behaviour. Arrows in panels for Affimer 14 indicate a motile single filament bundle. Arrows for Affimer 24 indicate accumulation of short actin filaments at the base of the lamella at 110 and 170 sec. Images captured using the Zeiss 880 LSM confocal using the Airyscan. Scale bar (2 μm) applies for all images.

To determine if we could see evidence for dynamic actin behaviour, we performed live cell time-lapse imaging for each of the three eGFP-tagged Affimers in B16 cells, capturing images every 10 seconds (Fig. 6, Supplementary Movies). All three eGFP-Affimers bound to actin filaments and bundles, and revealed elements of F-actin dynamics, but with distinct differences. eGFP-Affimer 6 revealed distinct populations of actin filaments that were stable or able to disassemble (Supplementary Movie 1). eGFP-Affimer 14 highlighted movements of shorter actin bundles but did not show their disassembly (Supplementary Movie 2). eGFP-Affimer 24 showed dynamic actin filament behaviour associated with the base of the lamella, in an area commonly associated with cell adhesion (Supplementary Movie 3). In cells transfected with the eGFP-Affimers, filopodia were generally short and not prominently labelled, in agreement with our observations in fixed cells (Fig. 4B). In contrast, F-tractin^20^ and Lifeact^16^ (Supplementary Movies 4 and 5) not only showed dynamic actin filament behaviour, but they also labelled filopodia, which were long and motile, particularly for cells expressing Lifeact. The reason for the reduced labelling of filopodia by the eGFP-Affimers is unclear but may be related to an inability of these Affimers to recognise the types of F-actin structures in filopodia.

### FRAP experiments demonstrate that Affimers 6, 14 and 24 have different lifetimes on F-actin

**Figure 7:**
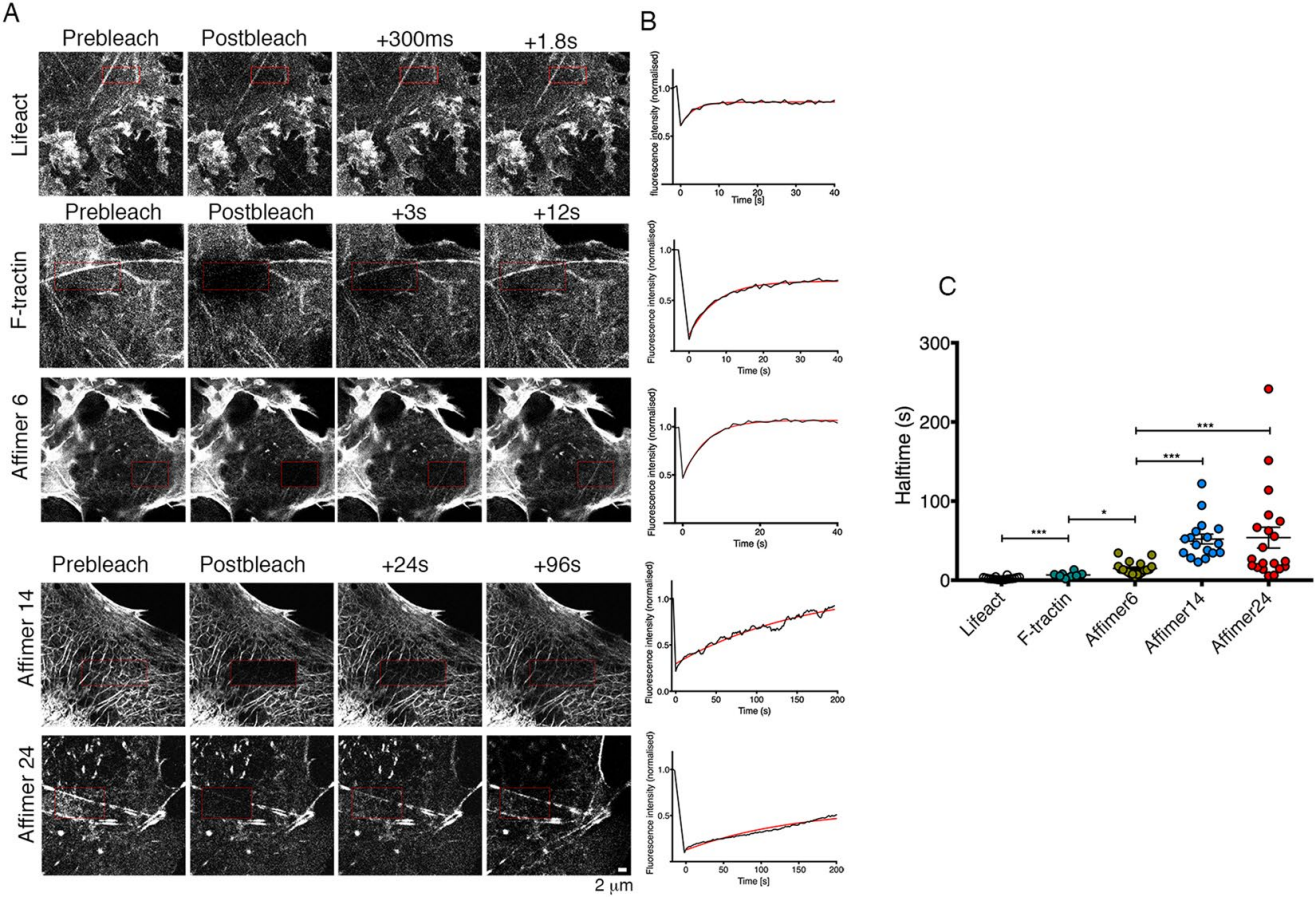
FRAP demonstrates that the eGFP-Affimers exchange onto F-actin at different rates (**A**) Example images pre- and post-FRAP of COS-7 cells expressing eGFP-Affimers 6, 14 and 24. COS-7 cells expressing Td-tomato F-tractin, or GFP-Lifeact were used for comparison. The red box indicates the region that was bleached. Supplementary movies for these images are provided (Supplementary Movies S6–10). Note that the example frames post-bleach for Lifeact are separated by much shorter intervals than for the remaining constructs, as fluorescence recovery was very fast for this construct. Images captured using the LSM 880 confocal. (**B**) example raw traces (corrected for background and any photobleaching) for the images shown in A, fit using a single exponential curve (superimposed as a red line). The data were fit using using Graph Pad Prizm. Scale bar (2 μm) applies to all images. (**C**) Show estimates of the half times for fluorescence recovery, from the exponential fits, for each of the Affimers, F-tractin and Lifeact. The mean +/− SEM is shown as bars superimposed onto the plots individual values. (N = 18, 8, 19, 17, 20 for Lifeact, F-tractin, Affimer 6, Affimer 14 and Affimer 24, respectively). Levels of significance between measurements are indicated on the graph. *P < 0.05, **P < 0.001, ***P < 0.0001.

To determine if the differences in behaviour of eGFP-Affimers were related to differences in their rates of exchange onto F-actin, we performed FRAP experiments (Fig. 7A) and compared these results for Affimers with those for F-tractin and Lifeact. The values for the half-time of fluorescence recovery obtained from a single exponential fit to the recovery curves, show that the recovery time for Lifeact is fastest (2.3 s ± 0.3 s, mean ± S.E.M, n = 18) and those for Affimer 4 and Affimer 24 are slowest (52 s ± 6 s and 53 s ± 13 s respectively, n = 17 and 20). The recovery half time for F-tractin was slower than that for Lifeact (6.7 s ± 1.2 s, n = 8), and that for Affimer 6 was intermediate (14.7 s ± 1.8, n = 19). Experimentally, it was more difficult to bleach Lifeact effectively, due to its very fast fluorescence recovery. Lifeact binds to both monomers and F-actin^16^, and this is likely to contribute to the rapid recovery we observed in FRAP experiments. Of all the Affimers, Affimer 6 behaves most similarly to F-tractin, consistent with its more similar behaviour in reporting on actin dynamics in the cell body of live cells. The increased half-times measured for Affimer 14 and 24 suggest that these Affimers may have the potential to affect actin dynamics, as reported for phalloidin, although their effects may be less marked, as the mean half times of ~50 s, are shorter than that reported for phalloidin (half-time ~400 s).

**Figure 8:**
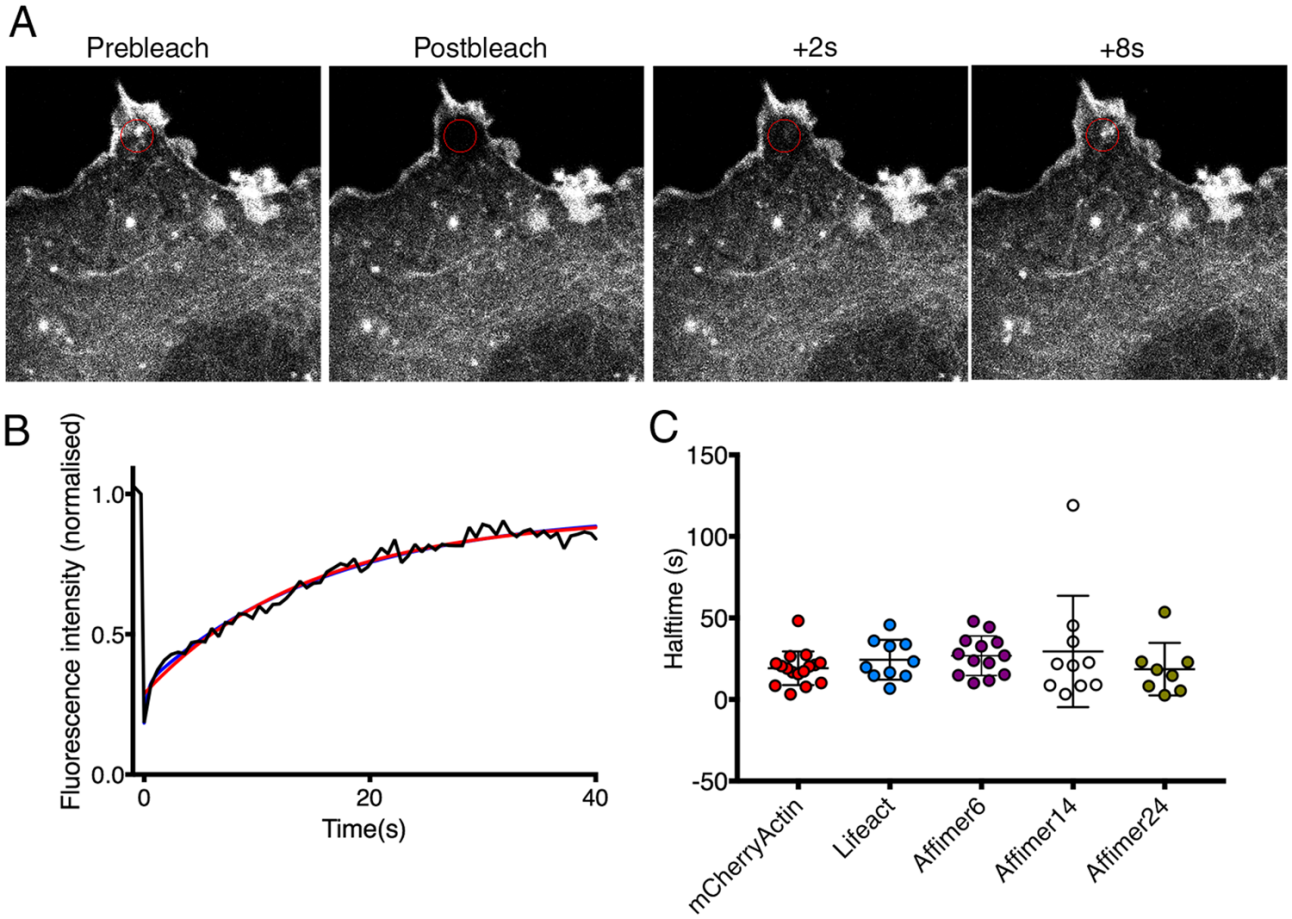
FRAP demonstrates that Affimers do not affect turnover dynamics of the actin cytoskeleton. (**A**) Example of a bleaching record for mCherry-actin in live COS-7 cells (and see Supplemental Movie S11). The red circle denotes the photobleached area. Scale bar (2 μm) as shown. (**B**) An example FRAP recovery curve for mCherry actin fluorescence recovery fit with a single exponential (red line) and double exponential (blue line). Fitted lines are almost identical. (**C**) Shows the values for τ measured from single exponential fits of mCherry-actin fluorescence recovery, bleached in the presence of eGFP-Lifeact, eGFP-Affimer 6, eGFP-Affimer 14 or eGFP-Affimer 24, or in their absence. T-tests were performed to determine any significant differences. None of the values were significantly different (N = 10 (mCherry-actin alone), and N = 10, 13, 10, and 8 for mCherry-actin recovery in cells co-transfected with Lifeact, Affimer 6, Affimer 14, and Affimer 24 respectively).

To determine if eGFP-Affimer expression affected actin dynamics, FRAP experiments on COS-7 cells co-expressing one of the eGFP-Affimers and mCherry-actin were performed essentially as described^33^ (Fig. 8), using circular regions of interest (ROIs), 2 μm in diameter. Cells co-expressing Affimer (or Lifeact) and mCherry-actin were identified, and mCherry-actin was bleached in those cells. The fluorescence recovery curves were fit using a single exponential, and the values obtained for τ is related to the dissociation rate, see reference^33^. Fitting with a double exponential did not significantly improve the fit to the data (Fig. 8B). None of the Affimers significantly affected the values for τ for mCherry-actin fluorescence recovery (Fig. 8C). These data suggest that actin dynamics are not affected by expression of Lifeact or any of the Affimers.

**Figure 9:**
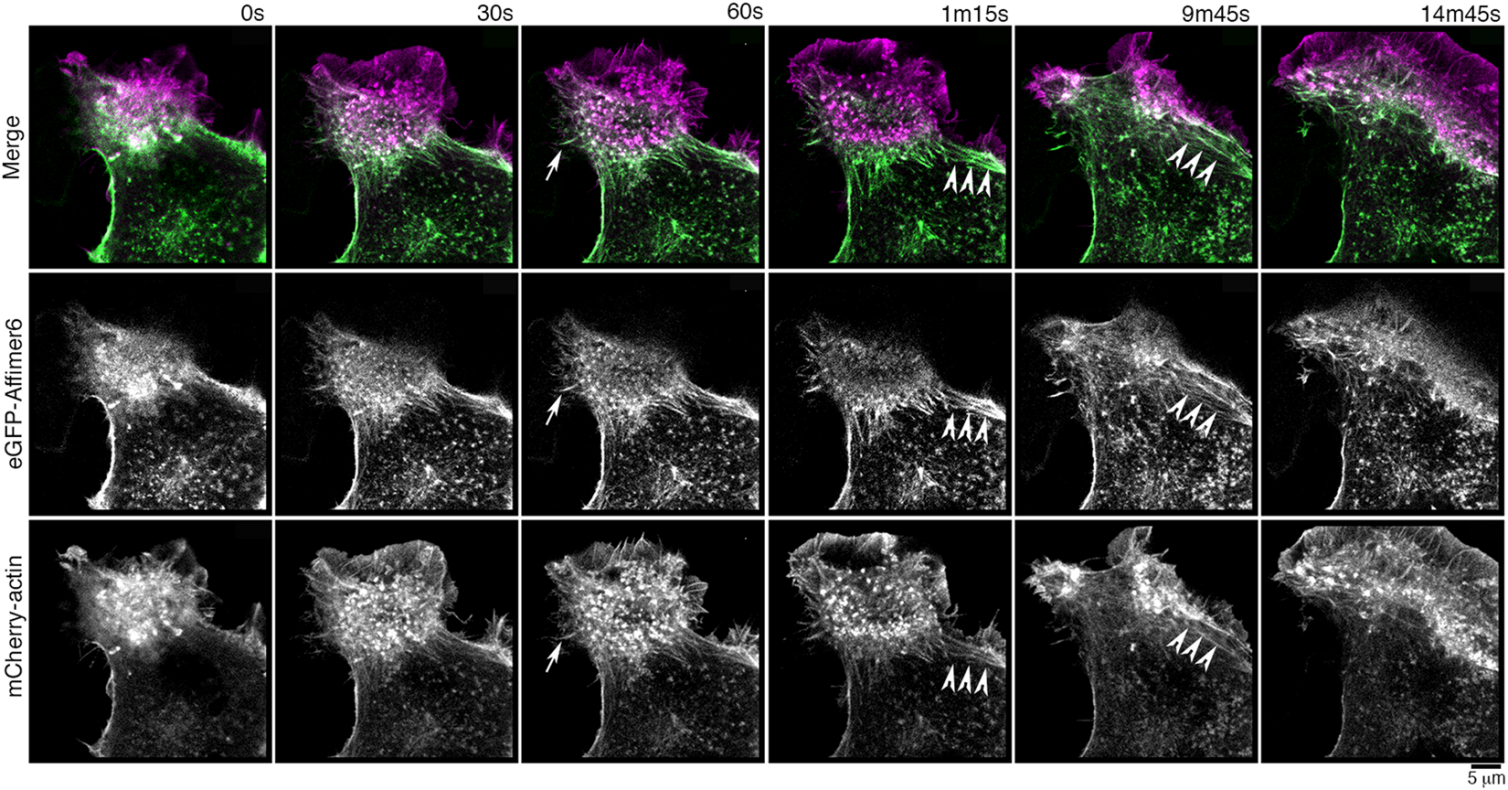
eGFP-Affimer 6 does not label the dynamic actin cytoskeleton in lamellipodia. Live cell imaging of B16 cells stimulated with PMA, 30 minutes before filming, showed extensive ruffling behaviour as evidenced by the mCherry-actin images (and see Supplemental Movie S12). eGFP-Affimer 6 was not recruited into these regions, but was co-localised with mCherry-actin on stable filopodial structures (arrowed) and stress-fiber like structures (arrowheads).

eGFP-Affimer 6 has properties most similar to those of F-tractin and Lifeact and does not appear to affect actin dynamics. To further explore its ability to report on actin dynamics, we co-expressed mCherry-actin and eGFP-Affimer 6 in B16 cells, and imaged them after stimulation with Phorbol-12-myristate 13-acetate (PMA), which increases protein kinase C activity, inducing ruffling behaviour^34^. Interestingly, we found that the Affimer was completely excluded from the highly dynamic ruffles (Fig. 9), and only co-localised with mCherry-actin in stress-fiber like structures (arrowheads, Fig. 9), numerous small puncta and some stable filopodial like structures (arrowed, Fig. 9). This suggests that Affimer 6 preferentially recognises and binds to more stable forms of F-actin. Similar experiments with Lifeact or F-tractin would be expected to show co-localisation with mCherry-actin in the ruffles.

**Figure 10:**
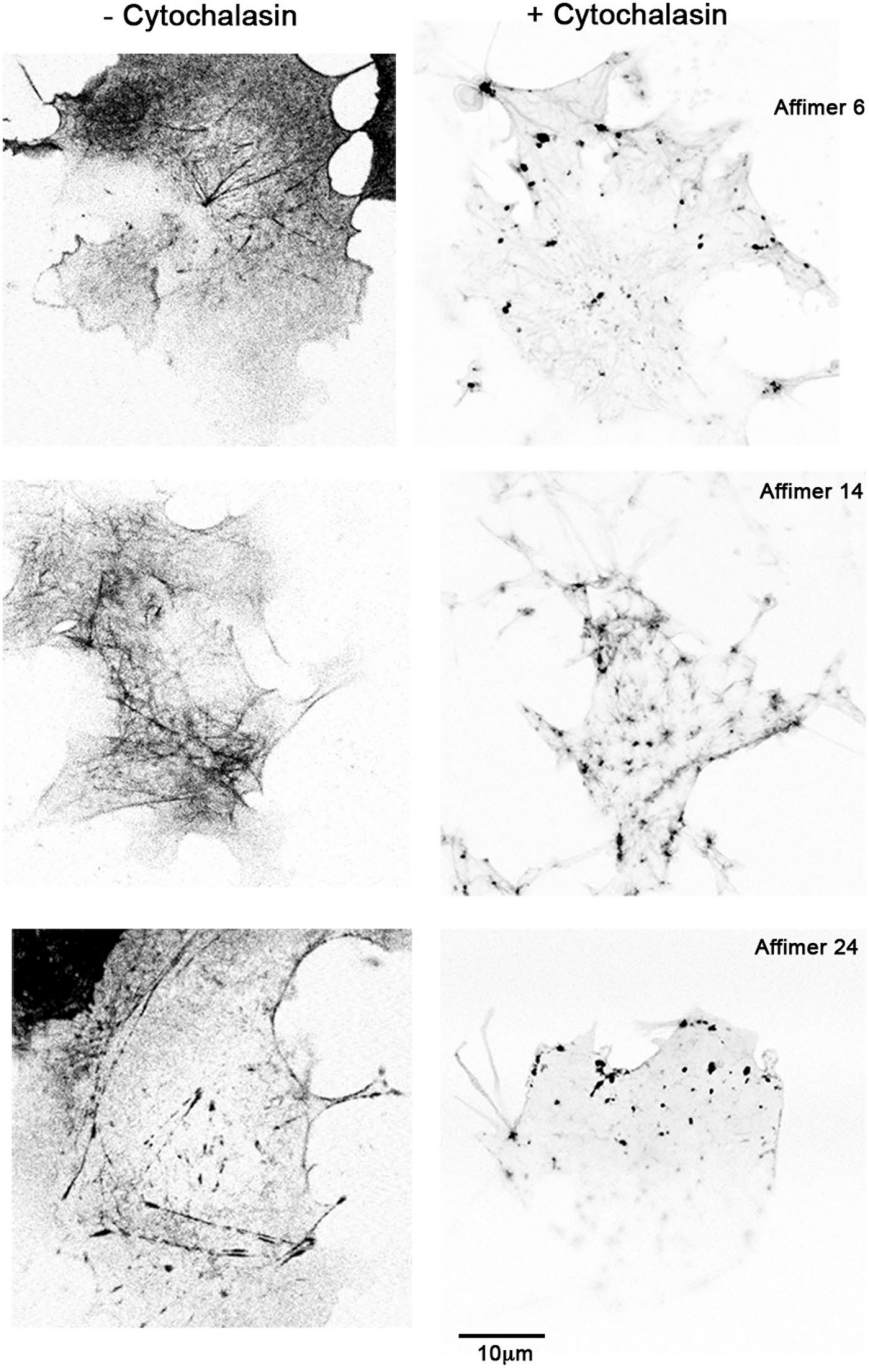
Actin filaments decorated with eGFP-Affimers are not resistant to depolymerization by cytochalasin D. Live COS-7 cells expressing eGFP-Affimers were incubated with 2.5 µM cytochalasin D for 20 minutes before imaging. Images captured using the Zeiss LSM 880 confocal. The images show that the actin filaments have mostly depolymerized, and small aggregates have formed.

Finally, to test if the eGFP-Affimers could protect actin filaments from depolymerization by the drug cytochalasin, we added cytochalasin D to live cells transfected with the eGFP-Affimer constructs (Fig. 10). We found that none of the Affimers protected the F-actin cytoskeleton from depolymerization. Thus, even though Affimers 14 and 24 may stabilize actin filaments as a result of their slow exchange rate, they do not affect the action of cytochalasin D.

### Affimer staining of F-actin in fixed cells depends on the method of fixation

**Figure 11:**
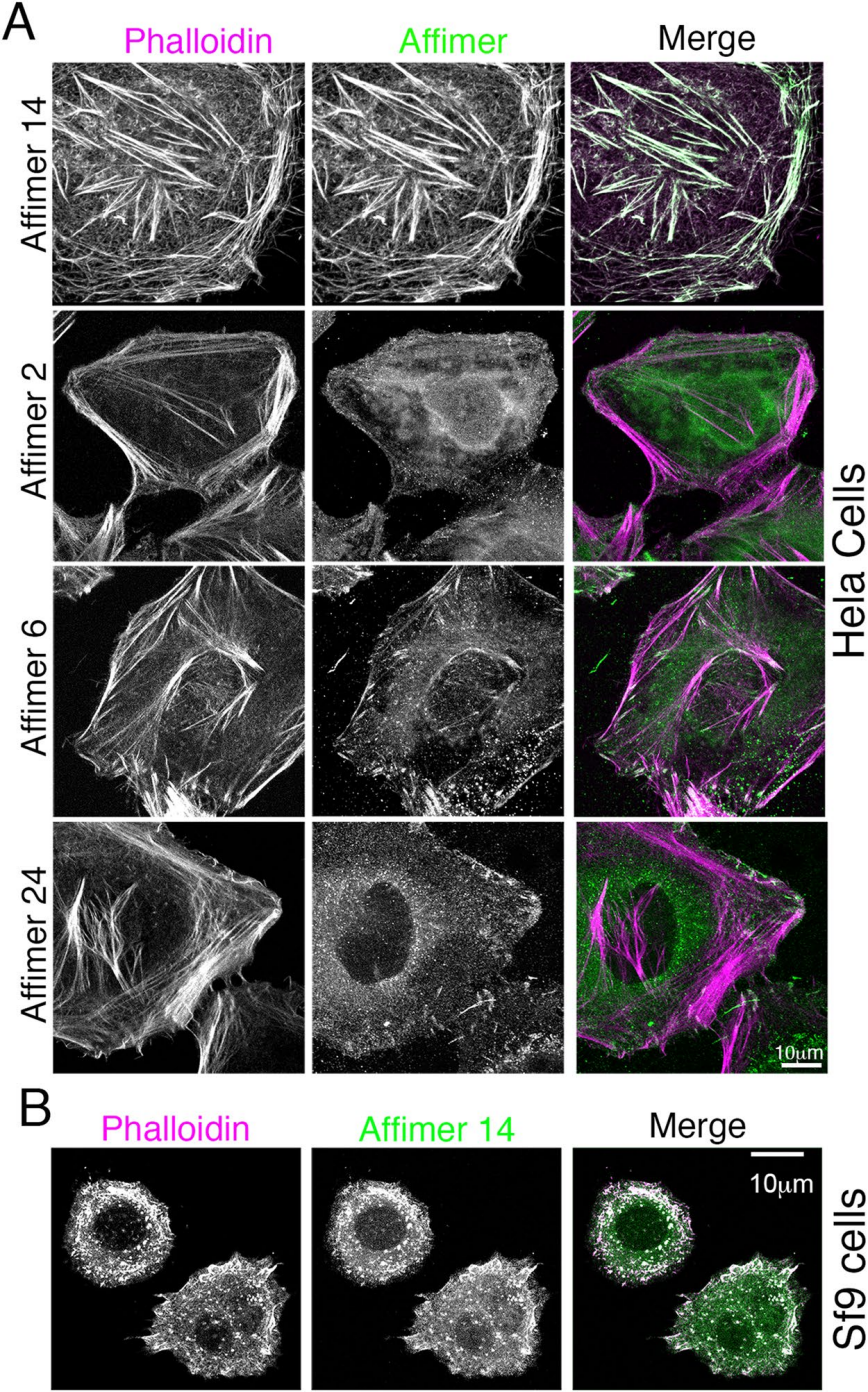
Testing the ability of purified Affimer to stain the actin cytoskeleton in paraformaldehyde fixed cells. (**A**) Hela cells were stained with each of the 4 different Affimers, using Affimers biotinylated on the C-terminal cysteine and visualized with Alexa 546 streptavidin (shown in magenta in merged image). Cells were co-stained with Alexa 488 phalloidin (shown in green in merged images). Scale bar as shown. (**B**) *Sf*9 cells were fixed and stained with Affimer 14, using a direct dye label (Alexa 488) on the C-terminal cysteine (shown in magenta) and co-stained with Alexa 546 phalloidin (shown in green in the merged image). Scale bar as shown.

**Figure 12:**
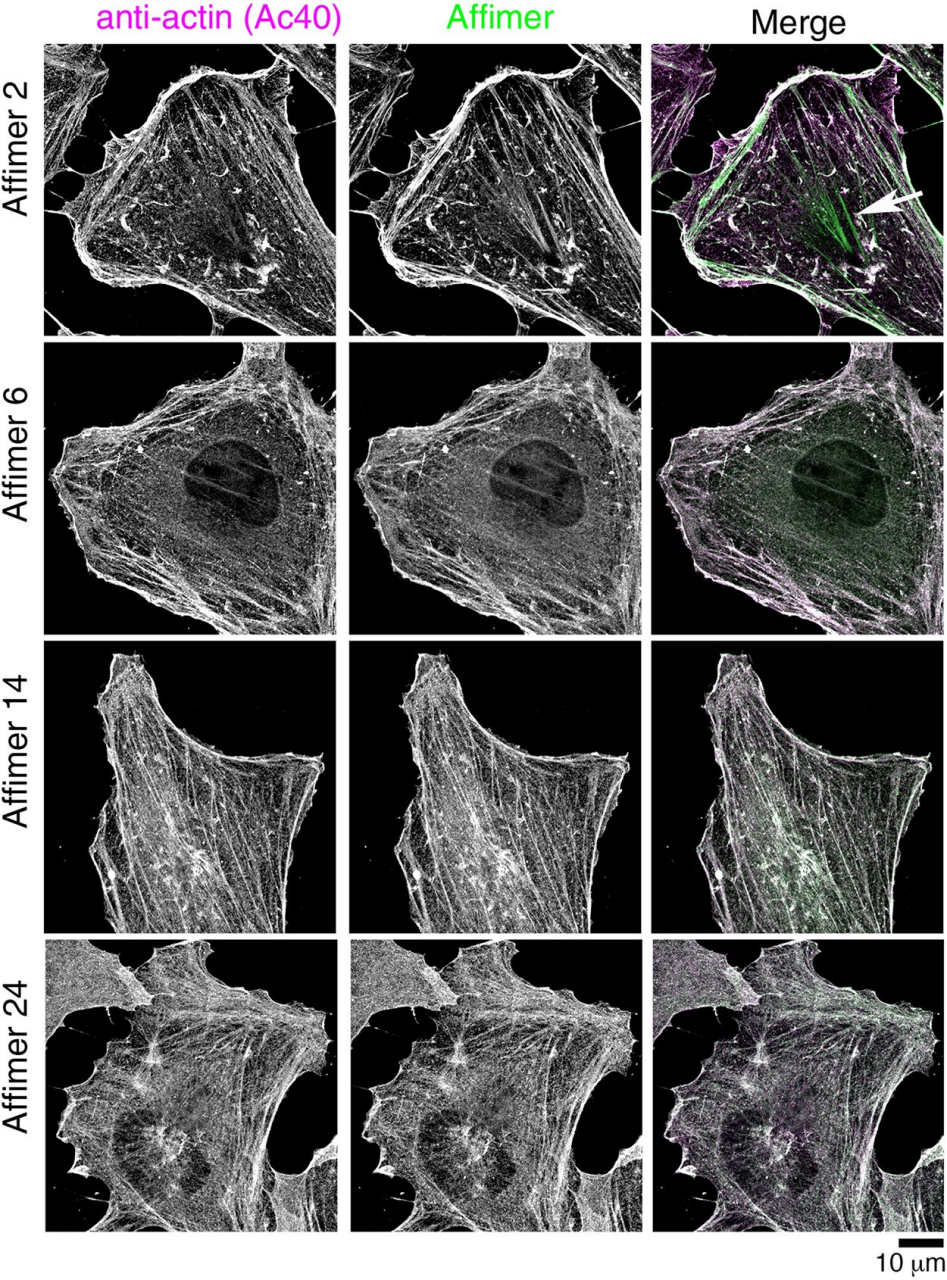
Testing the ability of purified Affimer to stain the actin cytoskeleton in methanol fixed cells. Hela cells were stained with each of the 4 different Affimers, using Affimers biotinylated on the C-terminal cysteine and visualized with Atto 488 streptavidin (shown in green in merged image). Cells were co-stained with an antibody to actin (AC-40), visualised with anti-mouse Alexa 546 antibody (shown in magenta in merged image). Co-localisation of magenta and green results in white. Scale bar as shown.

To determine if the Affimers could be used to label actin in fixed cells, we expressed and purified the Affimers, biotinylated the C-terminal cysteine, and determined if the purified protein was able to label the F-actin cytoskeleton in cells either fixed with paraformaldehyde or with methanol. These Affimers do not have a GFP tag, and their binding to actin is visualised using fluorescent streptavidin. In paraformaldehyde-fixed cells, only Affimer 14 specifically labelled the F-actin cytoskeleton (Fig. 11A). Staining for Affimers 2, 6 and 24 was mainly diffuse (Fig. 11). Co-staining for phalloidin showed that the staining pattern obtained for Affimer 14 and phalloidin were almost identical (Fig. 11A). Affimer 14 also labelled the actin cytoskeleton in paraformaldeyde-fixed insect (Sf9) cells (Fig. 11B). In contrast, in methanol-fixed cells, all 4 Affimers bound to and labelled F-actin (Fig. 12). Staining for F-actin with phalloidin requires the use of paraformaldehyde, as methanol, the other most commonly used fixative, alters the native conformation of F-actin and inhibits the binding of phalloidin. Therefore, in this case, an antibody was used to co-stain the cells for actin. It is interesting that even Affimer 2 labelled F-actin in this case, despite its apparent weak binding affinity for F-actin *in vitro*. It is possible that Affimer 2 preferentially binds to a specific conformation of F-actin, induced or stabilised by methanol fixation. Thus, aldehyde fixation interferes with ability of Affimers 2, 6 and 24 to bind to F-actin in fixed cells, but all 4 Affimers can bind to F-actin in methanol-fixed cells.

## Conclusions

Taken together, these data show that each of the Affimers labels F-actin in methanol-fixed cells, allowing them to be useful alternatives to antibodies to label F-actin when methanol fixation is required. Their small size (~2–3 nm) means that they should penetrate dense structures more easily than antibodies, as we observed for an Affimer for tubulin^24^, and they should also be useful for super-resolution imaging, as the labelled Affimer will place the dye molecule very close to F-actin, reducing the ‘linkage’ error. Purified Affimers are thus a strong alternative to phalloidin, which is not suitable for methanol-fixed cells, in labelling the F-actin cytoskeleton. They have the additional advantage that they can be expressed and purified from *E*. *coli*, and fluorescently labelled using the unique C-terminal cysteine, either directly to a dye, or indirectly through biotinylation. Of all the Affimers, Affimer 6 is probably the only one that appears suitable for labelling F-actin in live cells, and its preference for labelling stable actin filaments could be useful in probing actin dynamics.

The differences in labelling we observed in live cells between eGFP-labelled Affimers, Lifeact and F-tractin suggest that each of these constructs behaves differently. The differences in their behaviour may be linked to the differences in the rate of exchange of the Affimers, Lifeact and F-tractin from actin filaments, as demonstrated by FRAP experiments, and/or a preference for a specific subset of F-actin filaments. These experiments suggest that Affimers may have the ability to discriminate between small differences in F-actin structure. Further screening of the Affimer library against different actin isoforms or using negative-panning^28^ to isolate Affimers to different regions of actin could exploit this potential, generating new tools for investigating the F-actin cytoskeleton in the future.

## Materials and Methods

### Screening of the Affimer library by Phage Display

The library was screened as previously described^24,25^. Identification of actin-binding Affimers from the results of phage display were identified by phage ELISA. Briefly, 0.725 mg/ml biotinylated rabbit skeletal actin (Cytoskeleton) in phosphate buffered saline (PBS) was added to streptavidin-coated plates after first blocking with casein blocking buffer (Sigma-Aldrich). Growth medium and monoclonal Affimer phage reagents were added to each well. Bound phage was detected, after washing, with an HRP-linked anti-phage antibody (Seramun), and the binding visualized using TMB (3.3’,5,5’-tetramethylbenzidine) substrate, measured at a wavelength of 620 nm. Affimers that bound to actin were sequenced and Affimers with unique sequences taken forward.

### Subcloning of Affimers for expression in E. coli

The Affimer sequences were subcloned from the phagemid vector (pBSTG1^25^) into the pET11a vector using the NheI and NotI restriction sites. This additionally introduces a C-terminal His-tag for Ni-NTA resin affinity purification. In addition, each sequence was further subcloned to add a unique cysteine residue preceding the His-tag, to allow labelling in downstream experiments.

All the Affimers were expressed in BL21-CodonPlus(DE3)-RP *E*. *coli* competent cells (Agilent technologies). Cells from a single colony were grown innoculated into 5 ml 2YT medium (16 g/L Bacto tryptone, 10 g/L Bacto yeast extract, 5 g/L NaCl, pH7.0), supplemented with 1% w/v glucose and 100 μg/ml ampicillin. Cultures were incubated at 37 °C for 16–18 hours, while shaking at 230 rpm. The culture was then used to innoculate 250 ml of LB (Luria Broth) medium (KD medical), supplemented with 100 μg/ml ampicillin, and incubated at 37 °C, 230 rpm until the OD of the culture measured at a wavelength of 600 nm, reached 0.7 (usually 2.5 to 3 hours). The culture was induced with IPTG (isopropylthio-β-galactoside) to a final concentration of 0.1 mM and incubated at 25 °C, 150 rpm for 16–18 hours. Cells were harvested by centrifugation for 15 minutes at 3,500 *g*, and cell pellets stored at −80 °C prior to purification of the protein.

### Affimer purification

Cell pellets were resuspended in a 5 ml solution containing 4.5 ml lysis buffer (50 mM NaH_2_PO_4_, 300 mM NaCl, 20 mM imidazole, 10% glycerol, pH 7.4), to which 500 µl of 10× BugBuster (Novagen) and 5 µl Benzonase (25 unit/µl stock concentration, Novagen) was added. Resuspended cells were then incubated at room temperature for 30 minutes while shaking. To heat denature the low stability *E*. *coli* proteins, the samples were incubated at 50 °C for 20 minutes. Affimers are not affected by this step as they are stable at 50 °C. The suspension was then centrifuged for 20 minutes at 4 °C, and 3,000 *g*. The remainder of the purification steps were performed at room temperature. The supernatant was mixed with 1–1.5 ml of Ni-NTA resin (Biorad) in a 10 ml chromatography column pre-equilibriated with wash buffer (50 mM NaH_2_PO_4_, 500 mM NaCl, 20 mM imidazole, 10% glycerol, pH 7.4). The mixture was incubated for 1 hour, with occasional mixing. The resin was washed with 10–15 ml wash buffer and then incubated with 10 ml of elution buffer (50 mM NaH_2_PO_4_, 500 mM NaCl, 300 mM imidazole, 10% glycerol, pH 7.4) for 15 minutes before eluting the protein in 1 ml fractions. Fractions containing the Affimer were combined and dialysed using 7 kDa cut-off dialysis cassettes (Thermo Scientific) against PBS, pH7.4 containing 1 mM DTT for 16–18 hours at 4 °C). Cys-Affimers were biotinylated using biotin maleimide (Sigma), first reducing any cystines to cysteine using tris(2-carboxyethyl)phosphine (TCEP) disulphide reducing gel (Thermo Scientific). Equal volumes of Affimer in elution buffer and TCEP (pre-washed in PBS) were mixed for 1 hour at room temperature with rocking. The mixture was then centrifuged, biotin-maleimide was added to the supernatant to a final concentration of 0.5 mM, and the mixture incubated on ice for 4 hours. The biotinylated Affimers were then dialysed against PBS as above.

### Actin and myosin purification

Actin was prepared from rabbit skeletal muscle acetone powder (Pel-Freez Biologicals) as described previously^35^ and stored as F-actin in 4 mM MOPS pH 7.0, 2 mM MgCl_2_, 0.1 mM EGTA, 3 mM NaN_3_, and 1 mM DTT. Mouse myosin-5b subfragment 1 (S1) and rat calmodulin were produced in *Sf*9 insect cells, the complex purified via FLAG-capture and stored in 10 mM MOPS pH 7.0, 500 mM NaCl, 0.1 mM EGTA, and 2 mM DTT^36^.

### Actin spin-down assays

To determine the dissociation constant of the Affimers to F-actin, F-actin and Affimers were mixed and adjusted to a final volume of 200 µl that contained 2.5 µM F-actin and 1–10 µM Affimer in a buffer comprising 25 mM MOPS pH 7.0, 50 mM KCl, 2 mM MgCl_2_, and 1 mM DTT. After mixing, the mixture was incubated at room temperature for 30 minutes, before centrifuging at 4 °C for 1 hour, at 110,000 g, using a TLA100 fixed angle rotor. 50 μl of supernatant was mixed with 50 μl of 2× Laemmli buffer, and the pellet was dissolved in 200 μl of 1× Laemmli buffer, at a temperature of ~100 °C. 50 µl of the dissolved pellet were subsequently mixed with 50 µl 2× Laemmli buffer and equal volumes of the supernatant and pellet fractions were analysed by SDS-PAGE using 4–12% gradient NuPAGE gels (ThermoFisher). Three independent sets of binding assays were run for each Affimer, and the data shown is the average of three measurements together with the standard error.

### Affimer competition assays

To test if Affimers bind to the same, or overlapping sites on F-actin, F-actin and two different Affimers were mixed such that all three proteins were each at a final concentration of 2.5 µM, and the sedimentation assay repeated as described above.

To test if Affimers bind to the same, or overlapping sites on F-actin as myosin, F-actin, Affimer (diluted in phosphate buffered saline supplemented with 1 mM MgCl_2_) and the S1 fragment of myosin-5b (diluted from a stock in 500 mM NaCl) were mixed to have a final concentration of 2.0 µM each of F-actin and myosin S1, and 0.5–3.0 µM Affimer. We estimated the final salt concentration for these experiments to be ~200 mM. After mixing, the samples were incubated, centrifuged and analyzed as described above.

All gels were stained using 0.25% w/v Coomassie Brilliant Blue R-250, 10% v/v acetic acid and 50% v/v methanol) for 1–2 hours, and destained using 7.5% v/v acetic acid, 27.5% methanol for 12 hours. Destained gels were imaged with an Odyssey Imaging System (LI-COR biosciences) at a wavelength of 700 nm. Images of the gels were analyzed in ImageJ to generate the data for the binding curves. Affimer (or myosin S1) band intensities in the pellets were normalized by the intensities of actin to account for variations in recovery of actin together with bound Affimer in the pellet. Bound Affimer (or S1) was calculated, and the dissociation constants calculated from a quadratic equation as described^37^.

### Myosin S1 ATPase activity assay

To determine the effect of the Affimers on actin-activated MgATPase activity of myosin S1, ATPase activity assays were performed in 10 mM MOPS, pH 7.0, 1 mM ATP, 50 mM NaCl, 2 mM MgCl_2_, 0.15 mM EGTA, 40 units/ml L-lactic dehydrogenase, 200 units/ml pyruvate kinase, 0.2 mM DTT, 200 µM NADH, and 1 mM phosphoenolpyruvate at a protein concentration of 50 nM myosin-5b S1 in a total volume of 200 µl. The reaction mix was supplemented with 1–5 µM Affimers and 4 µM F-actin, so that not all myosin heads will be bound to F-actin. At this actin concentration, ATPase activity will depend on the available concentration of actin in a quasilinear manner. This way if Affimers occupy the binding site of the myosin motor on F-actin, the observed reduction in ATPase activity would reflect the reduction of concentration of available actin.

The measurements were started by adding ATP to the reaction mixture. Oxidation of NADH, which indirectly shows the rate of the ATPase reaction, was measured by following the fall in absorbance at 340 nm using a Cary Spectrophotometer. The experiments were carried out at 25 °C. Three experiments were performed, and the data were averaged. The measured activities were corrected for S1 activity measured without F-actin and for actin activity measured without S1.

### GFP-tagged Affimers

Affimer sequences were subcloned into the pEGFP-C1 mammalian expression vector (Clontech) by a PCR based approach. Sequences lacking the His-tag and cysteine residue were amplified and the resulting cDNA inserted into the EcoRI and BamHI restriction sites, downstream of and in frame with the eGFP coding sequence. mEGFP-Lifeact-7 was a gift from Michael Davidson (Addgene plasmid # 54610, derived from a 17 amino acid residue actin-binding peptide^16^ fused to the C-terminus of eGFP). Td-tomato F-tractin was a gift from Wolfgang Wagner (NIH) and consists of Td-tomato fused to the N-terminus of a 43 amino acid actin binding peptide derived from inositol 1,4,5 Trisphosphate 3-kinase A^18^).

GFP-tagged Affimers were transfected into COS-7, HeLa or B16 cells (ATCC). HeLa and COS-7 cells were cultured in DMEM (Dulbeccos Modified Eagles Medium) with GlutaMax (Thermo Fisher Scientific), and B16 cells were cultured in RPMI (Roswell Park Memorial Institute) medium. Both were supplemented with 100 units/ml of penicillin, 100 μg/ml streptomycin and 10% v/v FBS.

Cells were transfected with the eGFP-Affimer, Lifeact-7 or Td-Tomato-F-tractin expression plasmids using Fugene (Promega), following the manufacturer’s instructions. 24–48 hours later, they were either fixed and stained for further analysis, or the live cells were imaged at 37 °C. For live cell imaging, the normal culture media was exchanged for the same media, but lacking phenol red and with the addition of 10 mM HEPES, at least 2 hours before the experiment. To stimulate B16 cells, Phorbol-12-myristate 13-acetate (PMA) was added to a final concentration of 100 ng/ml, approximately 15 minutes before imaging. Imaging of fixed cells co-stained for phalloidin was performed using the Deltavision Deconvolution microscope, using the x63 1.4 N.A. objective lens. Further images were obtained using the Zeiss 880 LSM confocal equipped with Airyscan, either using the x40 1.4 N.A. objective or the x63 1.4 N.A. objective (for Airyscan).

FRAP experiments to investigate the rate of exchange of eGFP-labelled Affimers in live cells were performed as described^38^. Briefly, a small rectangular region of the cell, close to the cell membrane was bleached using 100% laser power (wavelengths of 488 nm for eGFP, or 561 nm for Td-Tomato), for a minimum of 20 cycles, or until the fluorescence signal had dropped to ~30% of the fluorescence level prior to the bleach. Images of the whole cell were then recorded at 1 frame per second, at low (<1%) laser power (to minimise bleaching), until fluorescence recovery in the bleached area had plateaued. Faster frame rates were used for Lifeact. The intensity of the bleached area was analysed using Zeiss FRAP software, which additionally performed background subtraction, and any photobleaching correction necessary, and normalised the data. The normalised data was imported into GraphPad Prizm and fit using either a one or two exponential function, and the fits compared. A visual comparison of the fits, together with the values for the residuals obtained gave us confidence that a single exponential fit was best for all of the data obtained. Two separate experiments were performed, with ~10 cells per experiment. Data with poor fits (low values of R^2^) were discarded.

FRAP experiments to investigate the effects of expressing Affimers on G-actin dynamics were performed essentially as described^33^. Cells co-expressing both Affimer and mCherry-actin (mCherry fused to β-actin, kind gift of Vic Small^39^) were identified. mCherry-actin was bleached with a circular ROI, 2 μm in diameter, at 80% laser power for 80 cycles. Bleach parameters were optimised as described^33^. All experiments were performed using a Zeiss 880 LSM confocal microscope, with cells at a temperature of 37 °C. FRAP data was recorded for ~10 cells per experiment, and two separate experiments were performed. Data was analysed as described above.

To determine the effects of cytochalasin, cytochalasin D (Sigma) was added at a concentration of 1 μg/ml (diluted 10,000× from a 10 mg/ml stock) to live cells, and cells incubated for 20–30 minutes before imaging.

### Staining of cells

Hela and *Sf*9 cells were cultured on 13 mm washed coverslips and then fixed using 4% fresh paraformaldehyde in PEM buffer (8 mM PIPES pH 6.8, 5 mM EGTA, 2 mM MgCl_2_). Alternatively, Hela cells were fixed in 100% methanol (from −20 °C stocks) for 10 minutes at 4 °C, followed by 10 minutes at room temperature. After fixation, cells were stored in PBS at 4 °C. Paraformaldehyde fixed cells were permeabilised using 0.1% Triton-X 100, and blocked with 1% BSA in PBS. Cells were labelled with biotinylated Affimers, diluted in PBS containing 1% BSA to a final concentration of 2.5 μg/ml, and incubated for 1 hour. The coverslips were then washed, fluorescently labelled streptavidin (ThermoFisher) at a dilution of 1/100 was added in PBS, and the cells incubated for 1 hour. Coverslips were washed in PBS, and coverslips mounted on glass slides in Prolong Antifade (ThermoFisher). Paraformaldehyde fixed cells were additionally co-stained for F-actin using fluorescent phalloidin (Molecular probes), or for non-muscle myosin 2B (Sigma).

For methanol fixed cells, a mouse monoclonal anti-actin antibody (AC-40, Abcam) was added at 1/100 dilution together with the diluted Affimer in the first incubation, and an Alexa dye-labelled anti-mouse secondary antibody (Thermofisher) was added in the second incubation at a dilution of 1/400.

## Electronic supplementary material

Description of Supplemental Movies

Movie S1

Movies S2

Movie S3

Movie S4

Movie S5

Movie S6

Movies S7

Movie S8

Movie S9

Movie S10

Movie S11

Movie S12
