# Supplementary material for: Affimer proteins for F-actin: novel affinity reagents that label F-actin in live and fixed cells
Source: Sci Rep. 2018 Apr 26;8:6572. doi: 10.1038/s41598-018-24953-4 (PMC5920084; doi:10.1038/s41598-018-24953-4)
Supplement: Supplementary file 1 — Description of Supplemental Movies [file 41598_2018_24953_MOESM1_ESM.docx]

Affimer proteins for F-actin: novel affinity reagents that label F-actin in live and fixed cells.

Anna Lopata, Ruth Hughes^1^, Christian Tiede, Sarah M. Heissler, James R. Sellers, Peter J. Knight, Darren Tomlinson^1^, Michelle Peckham^1^

Supplemental movies

Figure 6: FRAP. Supplemental movies 1-5. All movies were made from images captured using the Zeiss 880 LMS Airyscan at a frame rate of 1 frame every 10 seconds, for a total of 30 frames. Playback speed is 20 frames per second. Relative time is shown (top right) as min:sec:ms. Scale bar (shown top left) is 2μM.

Supplemental Movie 1: eGFP-Lifeact

Supplemental Movie 2: TdTomato-F-tractin

Supplemental Movie 3: eGFP-Affimer6

Supplemental Movie 4: eGFP-Affimer14

Supplemental Movie 5: eGFP-Affimer24

Figure 7: FRAP experiments for eGFP-Affimers. Supplemental movies 6-10. All movies were made from images captured using the Zeiss 880 LMS Airyscan. Playback speed is 20 frames per second. Relative time is shown (top right) as min:sec:ms. Scale bar (shown top left) is 2μM. Bleached area is indicated by the rectangle.

Supplemental Movie 6: eGFP-Lifeact

Supplemental Movie 7: TdTomato-F-tractin

Supplemental Movie 8: eGFP-Affimer6

Supplemental Movie 9: eGFP-Affimer14

Supplemental Movie 10: eGFP-Affimer24

Figure 8: Example mCherry-actin FRAP experiment. Supplemental movie 11. Movie made from images captured using the Zeiss 880 LMS Airyscan. Playback speed is 20 frames per second. Relative time is shown (top right) as min:sec:ms. Scale bar (shown top left) is 2μM. Bleached area is indicated by the red circle.

Figure 9: Time-lapse movie of B16 cells co-expressing mCherry-actin and eGFP-Affimer6, and stimulated with PMA. Supplemental movie 12. Movie made from images captured using the Zeiss 880 LMS Airyscan. Playback speed is 20 frames per second. Relative time is shown (top right) as min:sec:ms. Scale bar (shown top left) is 5μM. Magenta: mCherry-actin. Green: eGFP-Affimer6
